# Case series of ovarian neuroendocrine carcinoma: overview of clinicopathological features

**DOI:** 10.1186/s12905-023-02722-4

**Published:** 2023-11-13

**Authors:** Bao-Jie Feng, Tian-Hua Li, Yue-Hong Li, Jing-Xuan Feng, Jun Zhang, Jun-Ying Ma, Yan-Wei Wang, Su-Shuang Lu

**Affiliations:** 1 Department of Gynecology, the Fourth Hospital of Hebei Medical University, No.12 of Jian-kang Road, Chang-an District, Shijiazhuang, 050011 China; 2 Department of Pathology, The Second Hospital of Hebei Medical University, Shijiazhuang, 050000 China; 3 Department of Clinical Laboratory, The Second Hospital of Hebei Medical University, Shijiazhuang, 050000 China; 4 Department of Gynecology, The First Hospital of Hebei Medical University, Shijiazhuang, 050032 China

**Keywords:** Immunohistochemistry, Neuroendocrine carcinoma, Ovary, Pathological characteristics, Prognosis

## Abstract

**Background:**

Ovarian neuroendocrine carcinoma (O-NEC) is a relatively uncommon neoplasm, and the current knowledge regarding its diagnosis and management is limited. In this series, our objective was to provide an overview of the clinicopathological characteristics of the disease by analyzing clinical case data to establish a theoretical foundation for the diagnosis and management of O-NEC.

**Case presentation:**

We included three patients in the present case series, all of whom were diagnosed with primary O-NEC based on pathomorphological observation and immunohistochemistry. Patient 1 was a 62-year-old patient diagnosed with small cell carcinoma (SCC) of the pulmonary type. Post-surgery, the patient was diagnosed with stage II SCC of the ovary and underwent standardized chemotherapy; however, imaging examinations conducted at the 16-month follow-up revealed the existence of lymph node metastasis. Unfortunately, she passed away 21 months after the surgery. The other two patients were diagnosed with carcinoid tumors, one at age 39 and the other at age 71. Post-surgery, patient 2 was diagnosed with a carcinoid in the left ovary, whereas patient 3 was diagnosed with a carcinoid in her right ovary based on clinical evaluation. Neither of the cases received adjuvant therapy following surgery; however, they have both survived for 9 and 10 years, respectively, as of date.

**Conclusion:**

Primary O-NECs are rare and of diverse histological types, each of which has its own unique biological features and prognosis. SCC is a neoplasm characterized by high malignancy and a poor prognosis, whereas carcinoid tumors are of lesser malignancy and have a more favorable prognosis.

## Background

Neuroendocrine carcinoma (NEC) is a rare type of neoplasm in the female reproductive system, accounting for only 2% of gynecologic tumors. This neoplasm comprises a heterogenous group with varying biological potentials. The latest 2022 WHO description of the neuroendocrine tumor classification included neuroendocrine tumor of non-endocrine organs for the first time,and proposed a universal definition system of neuroendocrine tumor based on differentiation and proliferation classification .Epithelial neuroendocrine neoplasms are morphologically divided into well-differentiated neuroendocrine tumors (NETs) and poorly differentiated neuroendocrine carcinomas (NECs).And a tertiary classification system for the NEC classification was approved based on mitotic rate and proliferative capacity as assessed by KI67 index as well [1]. Primary ovarian neuroendocrine carcinoma (O-NEC) is rare, and its pathogenesis remains unknown. Due to limitations in understanding this disease and limited experience in pathology diagnosis, the current classification of NECs comprises carcinoid, atypical carcinoid, small cell NEC, and large cell NEC [1]. In this case series, we provide a comprehensive analysis of the clinicopathological data and follow-up of three patients who were diagnosed with primary O-NEC. Through an overview and analysis of the clinicopathological features and therapeutic progress of these tumors in the context of existing literature, we aim to enhance our understanding of their biological behavior and provide valuable insights for diagnosis and treatment.

## Case presentation

### Clinical data

Patient 1 was a 62-year-old female patient. A gynecologic ultrasound revealed a solid mass in the pelvic area, while a CT scan showed a cystic and solid mass in the same area, which was most likely from the uterus or ovary. The patient underwent a total hysterectomy, removal of the right adnexa and left ovarian tumor, left fallopian tube removal, greater omentum removal, appendectomy, and tumor reduction. Intraoperative findings: The patient had a normal-sized uterus with no right adnexal abnormalities. The tumor on the left ovary measured approximately 7 cm × 6 cm × 6 cm. The left ovarian tumor was surrounded by the sigmoid colon and attached to the posterior uterine wall. The tumor had invaded the left pelvic wall, pelvic floor, anterior rectal wall, and was attached to the posterior uterus wall up to the left sacral ligament. The left fallopian tube was dilated to approximately 1.5 cm above the pelvic inlet. Cancerous tissue had invaded the pelvic area around the ureter and adhered to the left pelvic wall.

Patient 2 was a 39-year-old female patient. Gynecologic ultrasound revealed a cystic and solid mass in the left ovary and a cystic mass in the right ovary. The patient underwent a laparoscopic resection of the left adnexa. Intraoperative findings: The patient had greater omental adhesions to the abdominal wall. After the adhesions were separated, the uterus returned to its normal size. The right fallopian tube was not seen, and there was an approximately 5 cm × 4 cm × 4 cm mass on the left ovary. The left fallopian tube and right ovary appeared normal.

Patient 3 was a 71-year-old female patient. Gynecologic ultrasound revealed a cystic and solid mass in the pelvic area, while a CT scan showed a similar lesion in the right adnexal region. The patient underwent a laparoscopic bilateral adnexectomy. Intraoperative findings: The patient was found to have uterine atrophy and a cystic mass measuring approximately 6 cm × 5 cm × 4 cm with a thin and smooth wall on the right adnexa. No other abnormalities were observed.

All specimens from the three patients were fixed using 4% neutral paraformaldehyde. Pathology slides were made as per routine procedures and observed under a microscope to determine the histopathological morphology of the tumors. Based on the results of routine pathological observations, immunohistochemical (IHC) investigations were performed for differential diagnosis. The antibodies used for immunohistochemistry included cytokeratin-pan (CKpan), carbohydrate antigen 125 (CA125), alpha-fetoprotein (AFP), carcinoembryonic antigen (CEA), Synaptophysin (Syn), Chromogranin A CgA, CD56, α-inhibin, caudal‑related homeobox transcription factor 2 (CDX2), Thyroid transcription factor 1 (TTF-1), and Ki67. The antibodies and IHC kits were purchased from Fuzhou Maixin Biotechnologies Co., Ltd.

### Results

#### Histopathology results

**Fig. 1:**
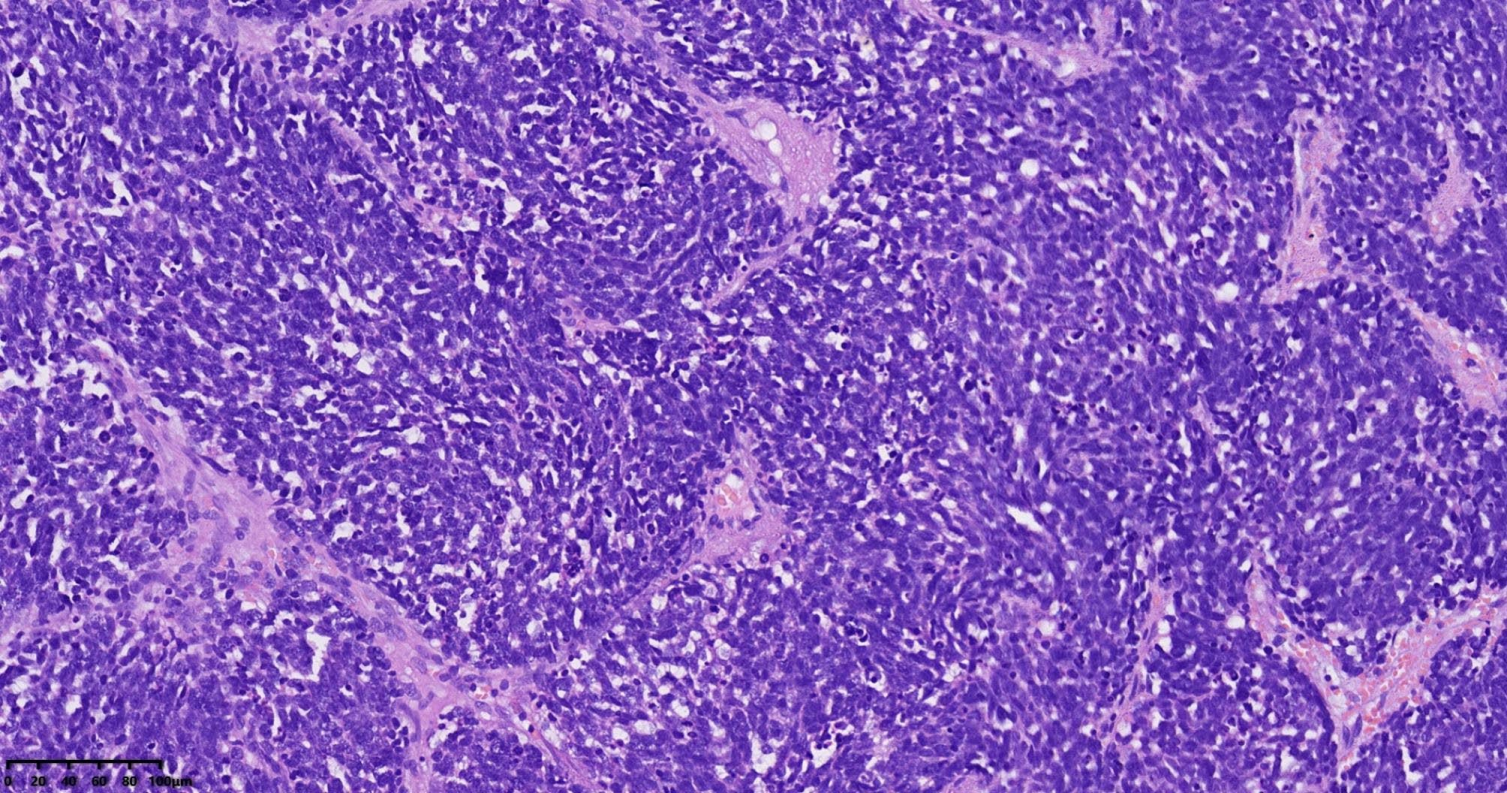
Small cell carcinoma (SCC) of the pulmonary type, Hematoxylin-Eosin Staining(HE) 200×

**Fig. 2:**
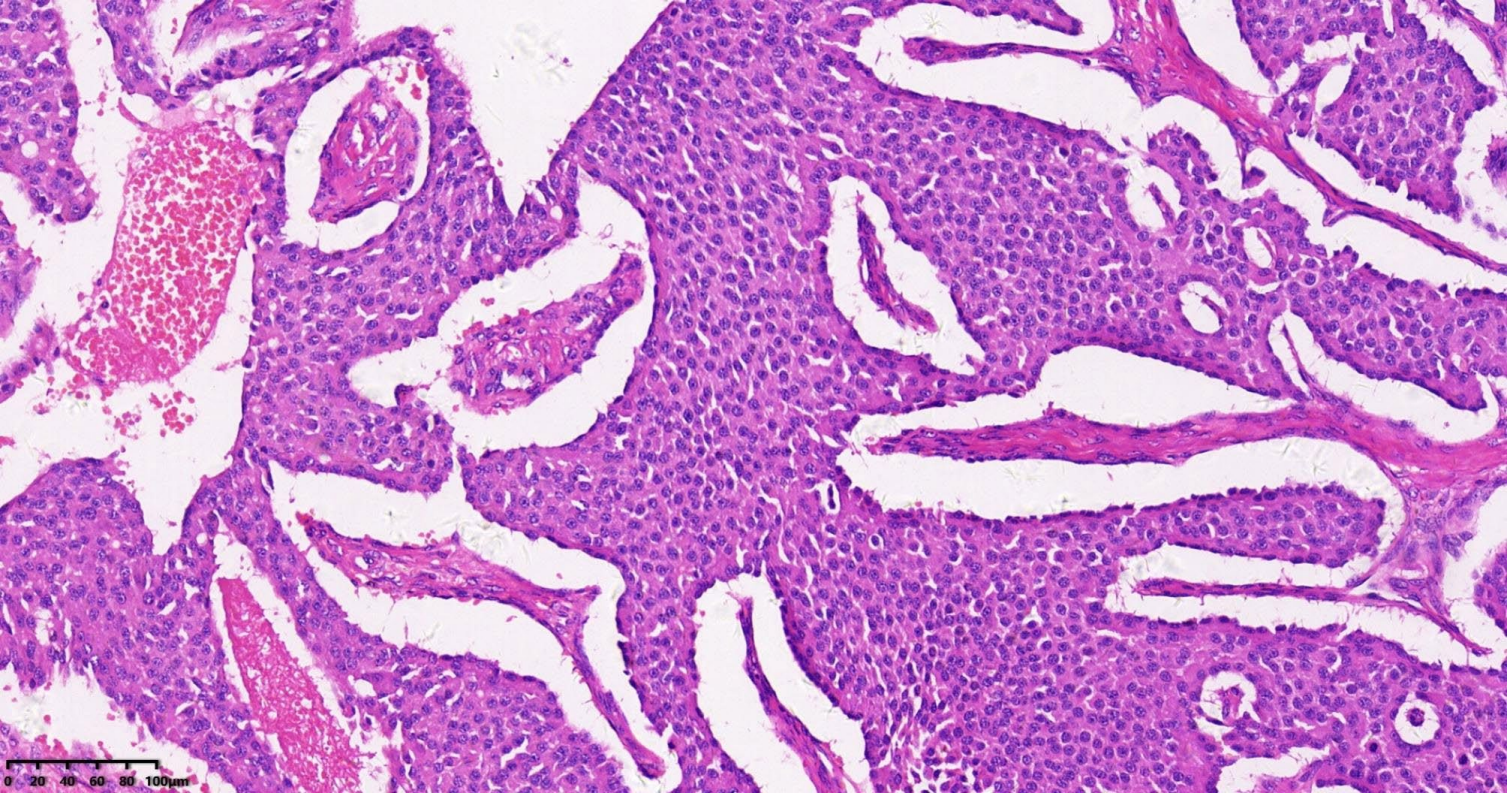
Carcinoid, Hematoxylin-Eosin Staining(HE) 200×

In patient 1, the tumor displayed a diffuse, patchy, nest-like distribution when viewed under an optical microscope. The tumor cells were small, with less cytoplasm and hyperchromatic nuclei. The nuclei were either oval or short spindle-shaped. There were numerous mitotic figures. There was evident necrosis (Fig. 1). In patients 2 and 3, the tumors exhibited distributions of small acinar, trabecular, stripe, and nest-like structures when observed under an optical microscope. The cells had a polygonal shape and consistent morphology, with less cytoplasm and hyperchromatic nuclei. The nuclei were either round or ovoid in shape and had finely speckled chromatin. There were few mitotic figures, and no foci of necrosis were observed (Fig. 2).

#### IHC results

**Fig. 3:**
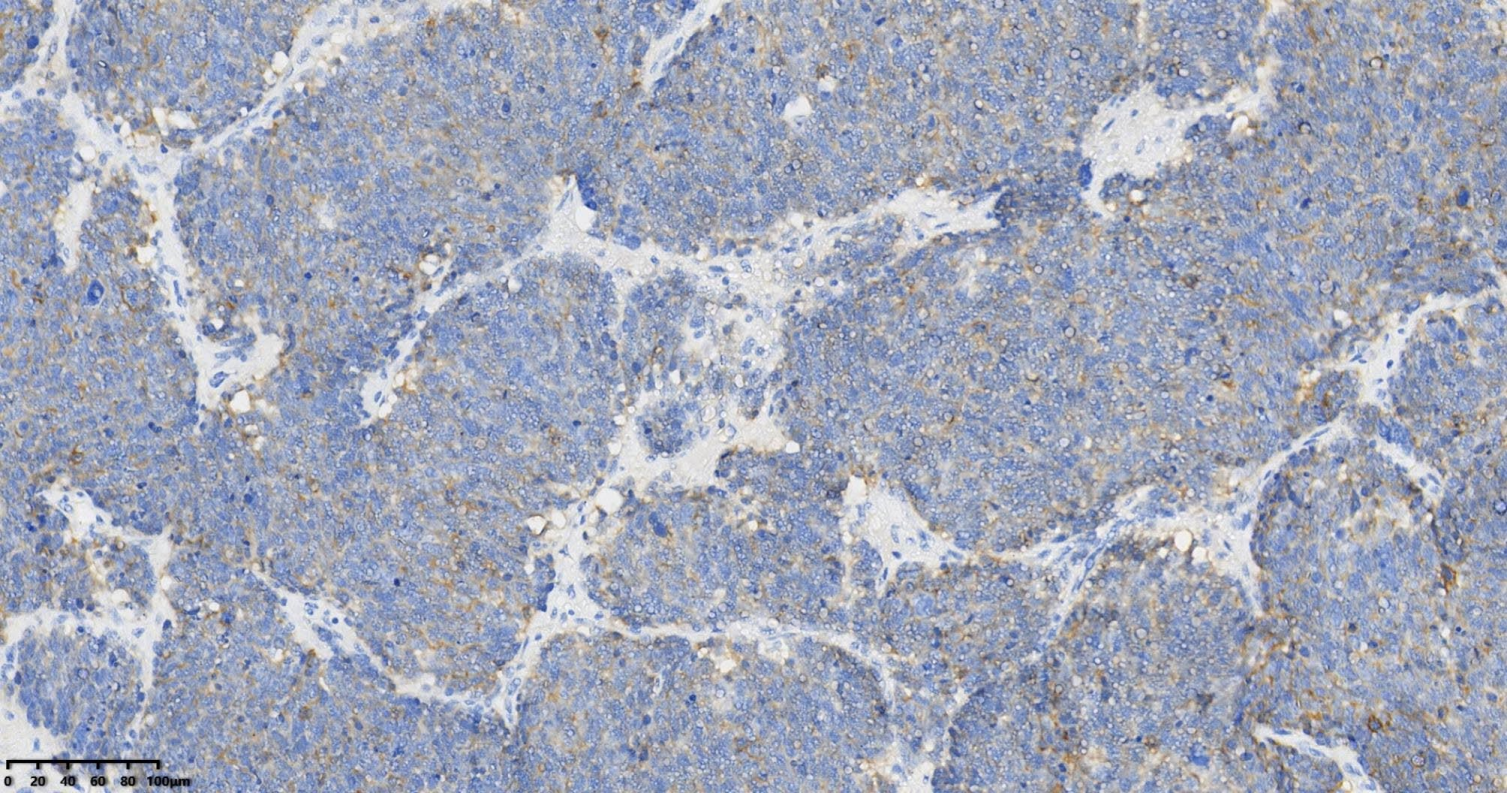
Small cell carcinoma (SCC), CD56 (+),Immunohistochemistry(IHC)200×

**Fig. 4:**
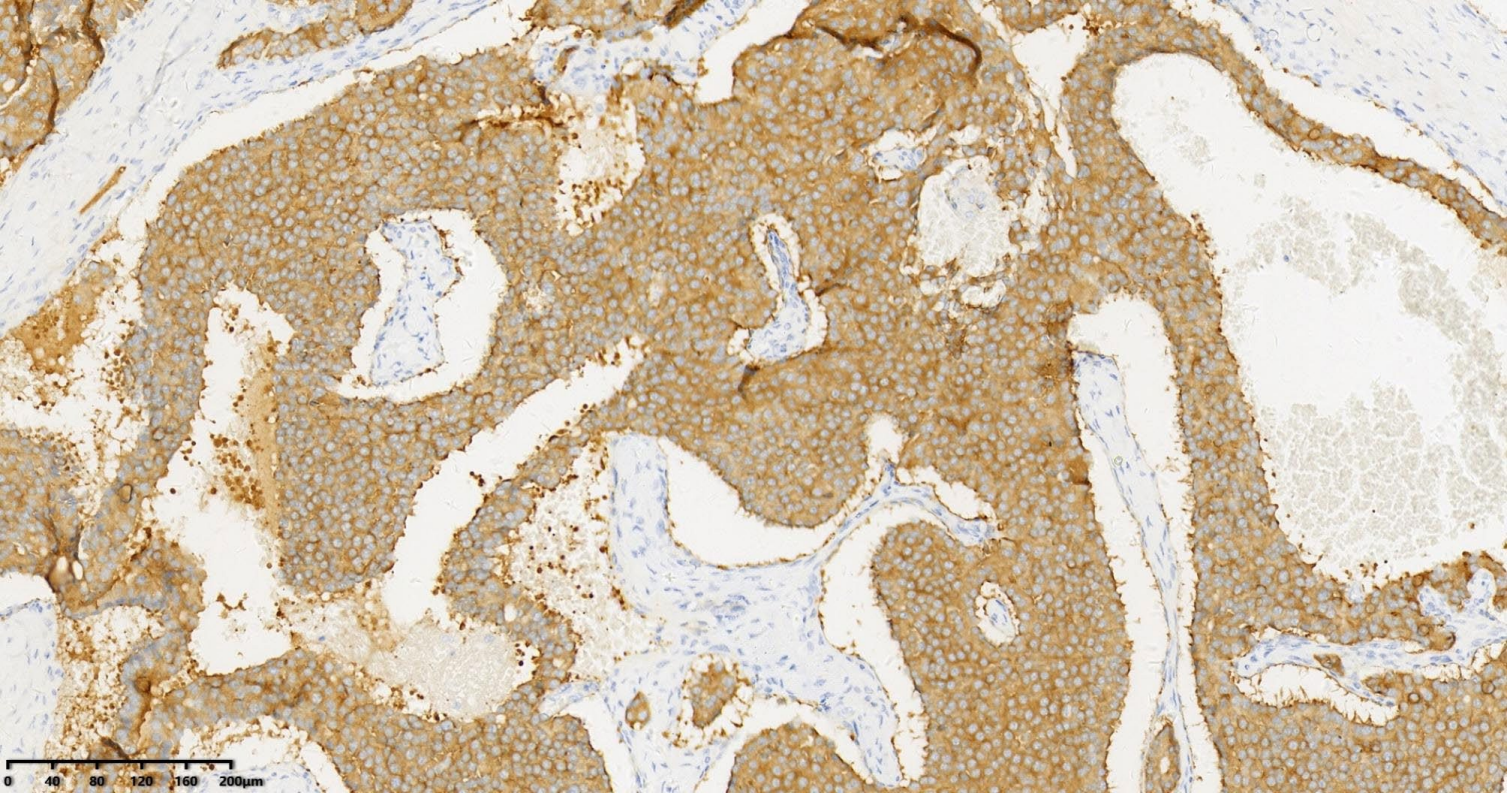
Carcinoid, SYN (+), Immunohistochemistry (IHC) 200×

Patient 1: CKpan (+), CA125 (-), AFP (-), CEA (-), Syn (-), CgA (-), CD56 (+), α-inhibin (-), CDX2 (-), TTF-1 (-), Ki67 (80%) (Fig. 3). Patients 2 and 3: CKpan (+), CA125 (-), AFP (-), CEA (-), Syn (+), CgA (+), CD56 (+), α-inhibin (-), CDX2 (-), TTF-1 (-), Ki67 (5%) (Fig. 4).

#### Pathology diagnosis results

The diagnosis of patient 1 was SCC of the left ovary with cancerous nodules present in the right parametrium, intestinal wall, and pelvic wall tissues. All other areas tested negative. The diagnosis of patient 2 was carcinoid of the left ovary. All other areas were normal. The diagnosis of patient 3 was carcinoid of the right ovary. All other areas were normal.

#### Treatment and follow-up results

Patient 1 was diagnosed post-surgery with stage II SCC of the ovary. After surgery, the patient received TC treatment (Taxol injection of 240 mg and Carboplatin of 0.5 g) every 3 weeks for a total of 8 courses. At the 16-month postoperative follow-up, CT, MRI, and PET examinations indicated the presence of multiple metastases in the abdominal lymph nodes, lumbar spine, and soft tissues. Three-dimensional conformal radiotherapy (3DCRT) with a dosage of 50 Gy/25 fractions was administered along with the radiosensitizer Actinomycin. The patient passed away 21 months after the surgery due to multiple metastases.

Patient 2 was diagnosed post-surgery with a carcinoid on the left ovary. The patient had fertility needs and did not receive any other treatments. To date, 9 years and 7 months since the initial follow-up, she has had no observed recurrences of metastasis in any subsequent tests. Patient 3 was diagnosed post-surgery with a carcinoid on the right ovary. The patient did not receive any adjuvant treatment after surgery and has been followed up for 10 years and 6 months to date without experiencing any recurrence or metastasis.

## Discussion and conclusions

The pathogenesis of primary O-NECs is not well understood. These tumors are primarily classified into three histological types: SCCs (hypercalcemic, pulmonary types), carcinoid (insular, trabecular, goiter, mucinous carcinoid, mixed types), and large cell NEC [1]. At present, there is limited clinical understanding regarding the incidence, histogenesis, treatment, and prognosis of this disease. In this report, we have described the various pathological features observed in patients with different pathological stages. We analyzed clinical history data, imaging data, and IHC results to summarize the prognosis of clinical treatment for these patients. Additionally, we analyzed long-term follow-up results to provide a reference for future clinical work and to improve patient prognosis.

Ovarian SCC is an extremely uncommon malignant tumor of the ovary. SCC (pulmonary type) is a subtype of ovarian SCC with neuroendocrine features. Patients with this type of cancer vary widely in age. The incidence of unilateral and bilateral ovarian tumors is almost equal. The tumor is predominantly solid, with a microscopic distribution of tumor cells arranged in nests or patches. The cells are smaller, have less cytoplasm, darkly stained nuclei, and inconspicuous nucleoli. The origin of the tumor is unclear, and its prognosis is poorly documented. Taraszewski et al. observed a postoperative survival time of approximately 18 months [2].

Patient 1 in this study was an elderly female with ovarian SCC (pulmonary type) who had already developed extra-ovarian involvement at the time of presentation. The microscopic pathological structural features were similar to the existing reports. The sample tested positive for CD56 and negative for Syn and CgA. Post-surgery, the patient received TC therapy (Taxol injection of 240 mg and Carboplatin 0.5 g) every 3 weeks for a total of 8 courses. The patient passed away 21 months after the surgery due to lymph node metastasis that occurred during the 16-month follow-up period. Ovarian SCC (pulmonary type) is a highly malignant tumor, and it is common to find extra-ovarian spread at the time of detection. Despite undergoing extensive surgical resection and receiving regular postoperative chemotherapy, the patient experienced rapid recurrence and extensive metastasis, ultimately resulting in death.

Most existing studies pertaining to O-NEC are primarily case reports. Cai et al. [3] reported a case of primary small cell O-NEC (pulmonary type) in a 65-year-old patient. A mass of approximately 20 cm in diameter was found in the pelvis. The tumor cells appeared lymphocyte-like, with scant cytoplasm and numerous mitotic figures under microscopy. IHC results: NSE (+), Syn (+), CgA (+), CD99 (+), Ki67 (70% +), Vimentin (-), LCA (-), CK (-), S-100 (-), CD10 (-), inhibin (-), CK7 (-), and EMA (-). Cancer infiltration was observed in the perimetrium, myometrium, endometrium, and serosal surface of both fallopian tubes, and it accumulated in the posterior wall of the bladder. The ultrasound revealed tumor recurrence during the 3-month postoperative follow-up.

Guo et al. [4] reported a case of a high-grade O-NEC (primary ovarian SCC, pulmonary type) in a 30-year-old patient. A 15 cm × 11 cm × 5 cm solid mass was found above the uterus. The tumor cells were arranged in an insular pattern. IHC results: CD56 (+), Syn (+), EMA (+), CgA (partially positive), Ki67 (70% +), CD99 (-), CK (-), Vimentin (-), Calretinin (-), α-inhibin (-), ER (-), and PR (-). The patient passed away four months after the surgery.

Niu et al. [5] reported a case of ovarian clear cell carcinoma with concomitant localized NEC in a 43-year-old patient. The patient had a cauliflower-like mass of approximately 13 cm in diameter with a poor and brittle texture, which was found in the right adnexa. IHC results: ER (focal +), PR (-), CgA (focal +), Syn (focal +), CD56 (+), Ki67 (30% +), CDX2 (+), CK (AE1/AE3) (punctate +), CK20 (focal +), CK7 (+). The final diagnosis was clear cell adenocarcinoma of the right ovary with an intestinal-type borderline mucinous tumor containing localized small cell NEC (probably pulmonary type). After the surgery, the patient underwent chemotherapy but passed away 12 months later during the postoperative follow-up period.

Primary ovarian SCC (pulmonary type) must be distinguished from metastatic SCC, granulosa cell tumors of the ovary, and primary or metastatic carcinoid tumors of the ovary. Metastatic SCC is morphologically identical to primary ovarian SCC. As a result, detecting the primary lesion mainly relies on medical history and relevant examinations. Ovarian granulosa cell tumors typically affect women of reproductive age and are usually encapsulated. The nuclei of the tumor cells exhibit folding or furrowing, resulting in a “coffee bean” appearance or the formation of Call-Exner vesicles under microscopy. Symptoms of hyperestrogenism related to the endocrine system are often observed in clinical practice. The immunophenotype expresses Vimentin, SMA, actin, and S-100 proteins but does not express EMA or NSE. In primary or metastatic carcinoid tumors, the morphology typically exhibits a rosette-like structure with atypical nuclei, few mitotic figures, and no evidence of tumor necrosis.

Primary ovarian carcinoid tumors are rare NECs that are often associated with teratomas. The tumors exhibit slow growth, low malignancy, and a low potential for recurrence [6]. Most patients with ovarian carcinoid tumors are typically asymptomatic, although some may present with carcinoid syndrome, which can include symptoms such as flushing, bronchospasm, abdominal distention, and constipation [7]. Tumors often develop unilaterally, vary in diameter, and can be yellow or yellow-brown in color. They may be either solid or cystic. Microscopically, tumor cells can have an insular, goiter, trabecular, mucinous, or mixed distribution. The most common types are insular and goiter-type ovarian carcinoid tumors. IHC testing for neuroendocrine markers, such as Syn, NSE, CgA, and AE1/AE3, can be helpful in making a diagnosis.

The two patients with primary ovarian carcinoid tumors in this study varied greatly in age. The tumors exhibited small acinar, trabecular, stripe, and nest-like distributions. The cells had a polygonal shape and were morphologically consistent, with less cytoplasm and hyperchromatic nuclei. The nuclei were either round or ovoid in shape and had finely speckled chromatin. There were few mitotic figures, and no foci of necrosis were observed. The cells were CD56 (+), Syn (+), and CgA (+). The systemic examination did not reveal any other abnormalities. The diagnosis of a primary ovarian carcinoid was made based on clinical, morphological, and IHC findings. The patients had a positive prognosis and were still alive as of the date of the follow-up period.

Zhao et al. [8] reported six cases of primary ovarian carcinoid tumors. The age of onset for the patients ranged from 28 to 54 years old, and the tumors occurred on the left and right sides of the ovary in three cases each. Microscopically, there were three cases of the trabecular type, two cases of the goiter type, and one case of the insular type. They had a low-grade malignancy with a favorable prognosis.

In the two cases of primary ovarian carcinoid tumors reported by Yu et al., [9] the age of onset for the patients was 35 and 48 years, respectively. There was one case each of the insular and trabecular types under microscopy. The tumor cells had a round or polygonal shape, and there was no evidence of recurrence observed during the 45- and 63-month postoperative follow-up periods, respectively. IHC showed that the tumor cells were Syn (+) and CK (+) to varying degrees, but α-inhibin (-), CD99 (-), TTF-1 (-), ER (-), and PR (-). The Ki-67 proliferation index was less than 1%. They had a good prognosis.

Nicolas et al. [10] reported a case of an ovarian carcinoid tumor combined with a teratoma. The patient was a 65-year-old woman with an enlarged right adnexa measuring 13 cm × 13 cm × 8.5 cm. Microscopic examination revealed nest and trabecular-type tumor cells with pleomorphic nuclei. IHC showed that the tumor cells were Syn (+), CD56 (+), chromogranin (-), and Ki67 (10–12% +). The patient had a positive postoperative recovery and was discharged after seven days of follow-up.

Two cases of ovarian carcinoid tumors were reported by Vora et al [11]. The age of onset for the patients was 40 and 26 years, respectively, and both had unilateral onset of symptoms. One of them also had a mixed teratoma as well. Under microscopy, the tumor cells had a nest or trabecular type with eosinophilic cytoplasm and finely speckled chromatin. IHC results showed that the tumor cells were Syn (+) and chromogranin (+) in two cases. The post-surgery disease-free survival ranged from 10 months to 12 years.

Primary ovarian carcinoid must be distinguished from metastatic carcinoid, small cell carcinoma, and granulosa cell tumor. The primary lesion of metastatic ovarian carcinoid is mostly in the small intestine, particularly in the ileum. Additionally, both ovaries may be involved. The microscopic appearance of the tumor is similar to that of a primary carcinoid tumor. However, it is not associated with ovarian teratomas. A thorough systemic examination can help identify the primary lesion. Although SCCs share similarities with carcinoid tumors in terms of cell morphology and arrangement, they can be distinguished by numerous mitotic figures and common necrosis. The nuclei of the ovarian granulosa tumor cells exhibit furrowing, resulting in a “coffee bean” appearance or the formation of Call-Exner vesicles under microscopy. Symptoms of hyperestrogenism related to the endocrine system are often observed in clinical practice. The immunophenotype expresses Vimentin, SMA, actin, and S-100 proteins but does not express EMA or NSE.

Treatment options for various types of primary O-NECs can vary. The current standard of care for patients with early-stage SCC is a total hysterectomy and bilateral adnexal resection. Patients in advanced stages undergo tumor cytoreduction, followed by postoperative adjuvant chemotherapy [12]. There are various chemotherapy regimens, such as PAC (cisplatin, adriamycin, and cyclophosphamide), VAC (vincristine, actinomycin D, and cyclophosphamide), and PVB (cisplatin, vincristine, and bleomycin), among others. The disease is highly aggressive, and the prognosis is poor even for patients diagnosed in the early stages.

The primary treatment for carcinoid tumors is surgical resection, particularly for stage I patients, which is highly effective in nearly 100% of cases. In contrast, the 5-year survival rate for patients with advanced carcinoid tumors is 33% [13]. Pathological results are crucial for assessing the nature and extent of tumors. Through pathological analysis, doctors can determine the grade of the tumor and whether there is cellular infiltration or metastasis. These results help in deciding whether adjuvant therapies such as chemotherapy or radiation therapy are needed and can provide a more precise evaluation of the scope and method of surgery. Laparoscopic surgery is a minimally invasive surgical approach commonly used in the treatment of ovarian neuroendocrine tumors. Compared to traditional open surgery, laparoscopic surgery offers significant advantages [14]. Firstly, it involves smaller incisions and the use of laparoscopic instruments, which helps reduce surgical trauma, postoperative pain, and shortens hospitalization and recovery time. Additionally, laparoscopic surgery provides better cosmetic outcomes, leaving smaller and more aesthetically pleasing scars. However, laparoscopic surgery also has limitations, including the requirement of high surgical skills and dependence on equipment. Compared to other treatment methods, laparoscopic surgery is generally considered a more direct and thorough treatment option, but the specific treatment plan should be determined based on the patient’s condition and the advice of the physician.

O-NECs are largely reported as isolated cases both in China and internationally, with no relevant treatment guidelines. However, SCC of the ovary is a rapidly progressing, highly malignant disease with a high mortality rate and a poor prognosis. Therefore, clinicians and researchers should pay particular attention to this disease. With the advent of new chemotherapeutic drugs, immunological breakthroughs, molecular biology, and biological and gene treatments, the therapeutic outcomes for this type of cancer are expected to improve significantly, leading to a better prognosis. Exploring the pathogenesis of the disease is an important aspect of future research on this tumor, as it can provide a basis for adjuvant therapy for patients with advanced disease.

In conclusion, there are several clinical types of O-NECs. The prognosis and survival of each type are determined by its diagnosis. Ovarian SCC is a highly malignant and invasive cancer that is prone to recurrence. In contrast, primary ovarian carcinoid is less malignant, less likely to metastasize, and patients have a better prognosis. Therefore, accurate pathological staging and early detection of primary O-NECs are crucial for improving patient survival [15, 16].

## Data Availability

Data related to the current study are available from the corresponding author on reasonable request.

## Abbreviations

NEC: Neuroendocrine
SCCO: Small Cell Carcinoma Of the Ovary
SCC: Small Cell Carcinoma
SCCOHT: Hypercalcemic Type Small-Cell Carcinoma
SCCOPT: Pulmonary Type Small-Cell Carcinoma

## References

[1] Rindi G, Mete O, Uccella S, Basturk O, La Rosa S, Brosens LAA, Ezzat S, de Herder WW, Klimstra DS, Papotti M, Asa SL (2022). Overview of the 2022 WHO classification of neuroendocrine Neoplasms. Endocr Pathol.

[2] Taraszewski R, Rosman PM, Knight CA (1991). Cloney DJ.Small cell carcinoma of the ovary. Gynecol Oncol.

[3] Cai J, Hua P, Hu ZM, Lin WY, Lai N. Primary small cell neuroendocrine carcinoma of ovary: a case report. J Canc Control Treat. 2014;(5):256–8.

[4] Guo HN, Li JJ, Zhang L, Ren X, Sun YX (2014). A case of ovarian neuroendocrine Tumor and literature review. Chin J Clin Obstet Gynecol.

[5] Niu H, Zhao LP, Liu Y, Wu SY, Xu TM. Clear cell carcinoma of ovary with intestinal borderline mucinous Tumor and small cell neuroendocrine carcinoma: a case report and literature review. Chin J Lab Diagnosis. 2016;(1):148–9.

[6] Feng Y, Xu H (2008). Primary ovarian carcinoid: a case report of 4 cases and literature review. China Health Care & Nutrition.

[7] Somak R, Shramana M, Vijay S, Nita K (2008). Primary carcinoid Tumor of the ovary: a case report. Arch Gynecol Obstet.

[8] Zhao JJ, Zhu HT (2016). Chinese Journal of Clinical and Experimental Pathology. Chin J Clin Exp Pathol.

[9] Yu SL, Xu JC, Liu DD, Li XQ, Wang QQ. Two cases of primary ovarian carcinoid and literature review.Hainan Med J. 2014;(22):3404–6.

[10] Orsi NM, Menon M. Primary ovarian neuroendocrine Tumor arising in association with a mature cystic teratoma: a case report. Gynecol Oncol Rep. 2016; (17): 83–5. 10.1016/j.gore.2016.07.003 PMC4971231 27508272

[11] Vora M, Lacour RA, Black DR, Turbat-Herrera EA, Gu X (2016). Neuroendocrine tumors in the ovary: histogenesis, pathologic differentiation, and clinical presentation. Arch Gynecol Obstet.

[12] Chen L, Dinh TA, Haque A (2005). Small cell carcinoma of the ovary with hypercalcemia and ectopic parathyroid hormone production. Arch Pathol Lab Med.

[13] Rabban JT, Lerwill MF, McCluggage WG, Grenert JP, Zaloudek CJ (2009). Primary ovarian carcinoid tumors may express CDX-2: a potential pitfall in distinction from metastatic intestinal carcinoid tumors involving the ovary. Int J Gynecol Pathol.

[14] Buzzaccarini G, Török P, Vitagliano A, Petousis S, Noventa M, Hortu I, Giannini A, Laganà AS (2022). Predictors of Pain Development after Laparoscopic Adnexectomy: a still open challenge. J Invest Surg.

[15] Pang L, Guo Z (2021). Primary neuroendocrine tumors of the ovary: management and outcomes. Cancer Med.

[16] Tullio Golia D’Augè, Giannini A, Bogani G, Dio CD, Antonio Simone LaganÃViolante Di Donato, Maria Giovanna Salerno, Donatella Caserta, Vito Chiantera, Enrico Vizza, Ludovico Muzii, Ottavia D’Oria. Prevention, Screening, Treatment and Follow-Up of Gynecological Cancers: State of Art and Future Perspectives. Clin. Exp. Obstet. Gynecol. 2023;50(8):160.

